# Therapeutic Pearls at Random Strung

**Published:** 1881-10

**Authors:** 


### THERAPEUTIC PEARLS AT RANDOM STRUNG.

### Treatment of Ozœna.

We observe in *L' Union Medicale du Canada* that chloral hydrate is highly recommended in chronic ozœna. It is used as an injection into the nose. The strength is as follows :

℞ Chloral Hydrate, . . . . . . ʒ ss.

Aquæ puræ, . . . . . . . ad. ℥ viij.

It is said to have cured cases of long standing which had been treated with a great number of remedies without benefit.—*Ex.*

### Treatment of Ozœna.

(*From La Tribune Medical*) for May 4th, 1879. Taken from the *Annales des Maladies de l' Oreille et du Larynx.*

Distend the nasal passages, when considerably narrowed by swelling of the mucuous membrane, and hard crusts which interfere with respiration, with graduated bougies, as in the urethra. Then use weak solutions of *Salicylate of Soda*, one part to five hundred of water, quite frequently, by means of Fauvel's retro-pharyngeal syringe. The parts being thoroughly cleansed in this way, Mappei applies *Calomel* to the surface by means of a nasal speculum.

PRURITUS OF THE VULVA.

℞ Sodii Boratis Pulv., . . . . . . ʒ j.

Plumbi Acet., . . . . . . . ʒ ss.

Tr. Opii., . . . . . . . ʒ i.

Aquæ Distil., . . . . . . ℥ viii. M. ft.sol.

S. When used dilute with one-fourth the quantity of warm water. Apply freely, on cloths saturated with this solution, to the external parts affected, and also between the labia—this to be repeated two or three times every fifteen minutes or twenty minutes. Then inject one ounce of solution into the vagina with a common glass syringe. This greatly ameliorated her sufferings. These applications were immediately followed by a free application of

℞ Glycerini, . . . . . . . ℥ i.

Acid carbolici, . . . . . . gtt. xx. M.

The relief was immediate and complete. The pruritus returned subsequently in a very light form twice, but was in each instance relieved immediately by the same remedies. About two years previous to the first attack, I treated the same lady for chronic inflammation and erosion of cervix uteri. Did this have anything to do with the pruritus ? This lady enjoys fair health, is regular in her menses, but has no children.

I treated a lady only a few months since with the same course,and a like result. This lady was pregnant, supposed to be in her third month, but otherwise enjoyed good health.

Atthill recommends in such cases, that the patient, after she has syringed or sponged herself with warm water, to lay inside the labia pieces of lint soaked in a lotion composed of carbolic acid grs. x., acet. morphia grs. viii., dilute hydrocyanic acid ʒ ii., glycerine ʒ iv. and water ℥ iv., M.

I have given the foregoing as additional sources and modes of relief in this very delicate, annoying and distressing affection. Of course, if the constitution was much depraved, the appropriate constitutional remedies should be applied.

Very respectfully yours,

E. MENDENHALL, M. D., *in Obstet. Gazette.*

Indianapolis, Ind.

### Rheumatism, Acute and Chronic, Hermicranid, &c.

℞ Guarana, grs. xv., with hot water, cream and sugar for a dose, and increase to *forty* grains once or twice a day.

Said to be *almost* a specific in Acute Rheumatism, and very beneficial in the cases above named.

### The Cyanides in Acute Rheumatism.

Dr. A. Luton gives the *Cyanide of Zinc* in pill, in doses of from three-fourths to one and a half grains in a single day.

The *Cyanide of Potassium*, pure and well prepared, is perhaps to be preferred, he thinks, to the Salt of Zinc, on account of its evident activity. In mixture he gives it in the dose of one and a half grains per day. It is best administered in the form of pills, coated with silver. It is not advisable to go beyond two grains a day.

